# Editorial: Drug resistance in maternal and paediatric bacterial and fungal infections: Is COVID-19 changing the landscape

**DOI:** 10.3389/fmicb.2023.1177669

**Published:** 2023-03-15

**Authors:** Felipe Piedade Gonçalves Neves, Tomislav Mestrovic, Tatiana Castro Abreu Pinto

**Affiliations:** ^1^Department of Microbiology and Parasitology, Instituto Biomédico, Universidade Federal Fluminense, Niterói, RJ, Brazil; ^2^University North, University Centre Varaždin, Varaždin, Croatia; ^3^Department of Health Metrics Sciences, University of Washington School of Medicine, Seattle, WA, United States; ^4^Department of Medical Microbiology, Instituto de Microbiologia Paulo de Góes, Universidade Federal do Rio de Janeiro, Rio de Janeiro, RJ, Brazil

**Keywords:** bacterial infections, fungal infections, pediatric diseases, maternal diseases, antimicrobial resistance (AMR), COVID-19

Globally, too many children under 5 years old and pregnant/postpartum women still dye every day from preventable causes, particularly in low- and middle-income countries (LMIC). In 2021, 5 million children under 5 years old died (~14,000 deaths every day). The under-five mortality, which is the probability a newborn would die before reaching 5 years of age, has been declining since 1990; however, it is still unacceptably high, reaching 38 deaths per 1,000 live births in 2021 (The United Nations Children's Fund, 2023). Deaths related to pregnancy and childbirth also affect a high number of women, with more than 800 deaths per day in 2017; the majority (94%) of them occurred in LMIC (World Health Organization, 2019).

A leading cause of under-five deaths is related to communicable diseases, including pneumonia (The United Nations Children's Fund, 2023), many of which can be successfully treated with antimicrobial agents or prevented with vaccines. However, drug-resistant microorganisms are emerging worldwide and represent a major global health threat (World Health Organization, 2017; Centers for Disease Control and Prevention, 2019).

Although antimicrobial resistance (AMR) develops naturally, the excessive and unwarranted use of antibiotics accelerates its emergence. The coronavirus disease 2019 (COVID-19) pandemic intensified the misuse of drugs—including antibiotics—with no evidence of effectiveness in the treatment of SARS-CoV-2 infection (Garg, 2021). Of note, SARS-CoV-2 infection during pregnancy/puerperium poses additional challenges (Metz et al., 2022). In this Research Topic of *Frontiers in Microbiology*, we highlight studies covering epidemiological and clinical features of maternal and childhood bacterial and fungal infections associated with AMR, affecting pregnant and postpartum women, neonates, infants and young children in the context of the COVID-19 pandemic. We also highlight vaccine development efforts against a major agent of bacterial pneumonia and present a review on a neonatal and multi-host bacterial pathogen from a One Health perspective.

Milenkov et al. present their results from healthy pregnant women in Madagascar as part of the human community component of the “Tricycle Project”—a WHO integrated global surveillance on ESBL-producing *Escherichia coli* embedded in the One Health approach—with an aim to sequence the whole genome of isolates obtained from human, food chain, and the environment (Hashim et al., 2022). The authors determined the carriage prevalence and risk factors associated with ESBL-producing *E. coli* colonization before the COVID-19 pandemic. They also performed several genetic characterizations of the isolates, which showed how IncY plasmids carrying a *bla*_CTX−*M*−15_ were found to be spread among ESBL-producing *E. coli* isolated from pregnant women in Madagascar. Notwithstanding a great diversity of clones disseminated throughout the country, SNP analysis based on whole genome sequencing revealed several genetically related isolates, suggesting human-to-human transmission.

Another bacterial species of relevance in the context of One Health, addressed by two works in this Research Topic, is *Streptococcus agalactiae*. Also known as Group B *Streptococcus* (GBS), this species is a major agent of pediatric disease in humans and an important pathogen of animals (Botelho et al., 2018). Oliveira et al. approach AMR in GBS under the One Health perspective and discuss the use of antimicrobial agents to control GBS disease, the evolution of AMR in the GBS population, and the future perspectives of resistant GBS infections in the post-COVID-19 era. As GBS can cause serious diseases in adults, Tulyaprawat et al. evaluated GBS isolates recovered from non-pregnant adults between 2017 and 2018 in Thailand and described the emergence of multidrug-resistant (MDR) isolates associated with non-serotype III isolates. Such isolates were resistant to tetracycline, clindamycin, and macrolides and their results suggest that these MDR isolates are closely related to the clonal complex 1.

Two other research endeavors evaluated possible changes in antimicrobial susceptibility among bacteria, during the COVID-19 pandemic. In Brazil, Pinheiro et al. conducted an observational retrospective cohort study to evaluate infections within a hospital from January 2019 to December 2021. Gram-negative bacteria predominated, with *E. coli* identified as the most common microorganism; however, *S. aureus* was the most frequent species among resistant bacteria. More specifically, infections with *S. aureus* were 3.1 times greater in pediatrics and maternal units than in other hospital wards. Still, no changes have been observed in the frequency of resistance profiles of the clinical isolates following the global emergence of SARS-CoV-2. Conversely, Ma et al. reported changes in molecular characteristics and AMR of invasive *S. aureus* infection isolates recovered from children in Kunming, China, although the annual incidence remained stable. From 2019 to 2021, the resistance to penicillin and sulfamethoxazole initially decreased and then increased, a trend that contrasted with the observed resistance to oxacillin, cefoxitin, erythromycin, clindamycin, and tetracycline. In addition, the dominant molecular type of methicillin-resistant *S. aureus* changed from ST338-t437 to ST59-t437 after 2019.

*Streptococcus pneumoniae* represents the major bacterial pathogen of community-acquired pneumonia, and the causative agent of other severe diseases, such as bacteremia and meningitis (Neves and Pinto, 2022). Several pneumococcal conjugate vaccines (PCV) are available to immunize children and adults against the most common serotypes associated with pneumococcal diseases (da Silva et al., 2023; Micoli et al., 2023), but Zhao et al. evaluated the immunogenicity and safety of a novel 13-valent PCV (PCV13) in healthy Chinese infants and toddlers. The major difference in this newly developed PCV13 is that each serotype is conjugated to a tetanus toxoid instead of the CRM_197_ of the original PCV13. They evaluated 1,040 healthy subjects and the novel vaccine was immunogenic for all serotypes, with a safety profile comparable to the 7-valent PCV.

Finally, Pinto et al. report the detection of an unusual etiologic agent of human mastitis, *Candida guilliermondii*, isolated from the milk of a puerperal woman with subacute mastitis in Brazil. *Candida* species can cause human diseases, ranging from mild oral and vaginal yeast infections to severe invasive diseases, and many of them are resistant to the antifungal drugs (Centers for Disease Control and Prevention, 2019). However, the patient had a full recovery after antifungal therapy.

In summary, we present new knowledge on epidemiology, clinical features, host-pathogen interactions and vaccine development against maternal and/or childhood bacterial and fungal pathogens, mostly associated with drug resistance, before and after the emergence of COVID-19. Of note, all microorganisms covered here are included in the CDC's AMR threats in 2019 in the USA (Centers for Disease Control and Prevention, 2019).

## Author contributions

All authors listed have made a substantial, direct, and intellectual contribution to the work and approved it for publication.
